# Current research trends on cognition, integrative complexity, and decision-making: a systematic literature review using activity theory and neuroscience

**DOI:** 10.3389/fpsyg.2023.1156696

**Published:** 2023-09-14

**Authors:** Isaac Molina, Edmundo Molina-Perez, Fernanda Sobrino, Mario Arturo Tellez-Rojas, Hilda C. Zamora-Maldonado, María Plaza-Ferreira, Yessica Orozco, Victor Espinoza-Juarez, Luis Serra-Barragán, Adolfo De Unanue

**Affiliations:** ^1^School of Government and Public Transformation, Tecnologico de Monterrey, Mexico City, Mexico; ^2^Faculty of Higher Education Iztacala, National Autonomous University of Mexico, Mexico City, Mexico

**Keywords:** cognition, integrative complexity, decision-making, activity theory (AT), systematic review

## Abstract

**Introduction:**

This article presents a systematic literature review that follows the PRISMA and PICOS guidelines to analyze current research trends on cognition, integrative complexity (IC) (a cognitive feature focusing on information processing in a person’s response rather than its quantity or quality), and decision-making from the perspectives of activity theory and neuroscience.

**Methods:**

The study examines 31 papers published between 2012 and 2022 and 19 articles specifically related to neuroscience. We performed a content analysis using six categories within activity theory: subjects, objects, rules, community, division of labor, and outcomes.

**Results:**

The study investigates the relationship between decision-making outcomes and IC as a cognitive feature in various contexts. Additionally, content analysis on neuroscience and IC revealed significant research gaps, including understanding the nature of IC, challenges related to its measurement, and differentiation from other cognitive features. We also identify opportunities for investigating the brain’s activity during decision-making in relation to IC.

**Discussion:**

We address the need for a more precise categorization of IC in studies of cognition, IC, and decision-making. We discuss the implications of our analysis for understanding the cognitive nature of IC and the potential of neuroscience methods for studying this attribute.

## 1. Introduction

### 1.1. Cognition and integrative complexity

The study of cognition, integrative complexity (IC), and decision-making has garnered significant attention due to their influence on behavioral decisions. IC is a cognitive feature that constructs a causal structure among various agents’ perspectives and data sources, integrating them into a coherent decision judgment for crisis resolution (Suedfeld et al., 1992). It focuses on processing information in a person’s response rather than its quantity or quality. IC finds application in diverse scientific fields, including political science and health, where it is employed to explore decision-making styles (Shao et al., 2019; Zhou et al., 2020) and differential diagnostics (Suedfeld, 2010; Conway and Woodward, 2019).

However, research on IC has become expansive, accompanied by various tools employed to study it. While contemporary authors such as Conway et al. (2018) and Zhou et al. (2021) utilize IC in their research, its definition and scope remain broad. This article aims to review IC research systemically, employing activity theory (AT), neuroscience, and the PRISMA and PICOS guidelines. These methodologies seek to comprehend research as a human endeavor with multiple components and actors interconnected. Additionally, this article addresses the inputs from theoretical and experimental research. It focuses on integrating IC as both a cognitive feature and a research variable, examining its implications and relationship with other cognitive features (e.g., intelligence, memory) as well as its application to various phenomena such as decision-making, organizational behavior, and politics.

Initially, IC was introduced as a cognitive feature associated with perception, information search, and decision-making (Driver and Streufert, 1969). It centers not on the quantity or quality of information an individual processes but on the information-processing systems of individuals and groups. The premise is that certain systems exhibit more complex information-processing capabilities than others, despite having the same inputs.

Integrative complexity encompasses two main components: differentiation and integration (Tetlock, 1986; Tetlock et al., 1993; McCullough, 2019). Differentiation pertains to the varying inputs from sources or the nature of available information used by an individual from different sources or of different natures. Integration refers to how these inputs are connected or synthesized rationally from an individual’s perspective. IC is measured on a 7-point scale, where a rating of 1 signifies no differentiation and integration, and a rating of 7 represents high differentiation and integration. Differentiation can range from emergent (2) to stable (3–7), while integration can be absent (1–3), emergent (4), stable (5), emergent at an important level (6), or highly integrated (7). Both components are present at each point and are measured in conjunction (Suedfeld et al., 1992).

Integrative complexity and decision-making are closely intertwined in research. IC analysis involves examining verbal inputs, such as speeches or interviews, and evaluating their differentiation and integration features. This makes IC valuable in assessing decision-making in various documented phenomena (Suedfeld et al., 2010). For instance, Suedfeld and Bluck (1988) reviewed public statements (proceedings of the UN Security Council, speeches from high-level officers to their parliaments, the public, or the media) during nine international crises that culminated in a surprise attack. They discovered that attackers exhibited a decline in complexity as the attack approached, indicating the potential of IC as a predictor of imminent strategic surprise. Furthermore, Wong et al. (2011) examined 61 Fortune 500 companies. They found that IC is a powerful management tool associated with corporate performance, particularly in decentralization, gathering information, and addressing stakeholders’ needs. Using a Q-sort methodology that synthesizes the information into concrete statements (Peterson et al., 1998), they analyzed IC and decentralization in strategic decision-making, correlating this with information on corporate social performance from the Kinder, Lydenberg, and Domini Socrates database. Their findings suggest that sociocognitive factors, such as IC, impact group decision-making.

### 1.2. Rationale and study objectives

The research on IC and its role in decision-making remains inconclusive. Békes and Suedfeld (2019) synthesized major findings on IC, deducing that it is more tightly linked to personality traits than cognitive abilities. They also found a weak association between intelligence and IC. It is important to note that a higher level of IC does not guarantee better decision outcomes, as IC interacts with situational, task, and material conditions in the decision-making process. Simple tasks require less IC compared to complex and demanding tasks. The level of IC is also closely associated with political views, with center-left-oriented individuals exhibiting higher levels of IC than those with more extreme left or right orientations. Furthermore, IC levels generally increase during periods of political tension escalation and decrease during resolution phases of political violence. However, it should be noted that all the evidence supporting these statements predates 2012, and IC remains an active concept in current research.

Assessing IC poses challenges as it deals with verbal data that often defies straightforward hierarchical organization. The assessment of IC prioritizes structure over content quantity and requires the judgment of at least two trained coders, who may concur or differ in their evaluations. A descriptive manual, such as the one provided by Baker-Brown et al. (2008), is used by judges to evaluate IC. This manual specifies each point on the 1–7 scale of IC and provides explanations, critical indicators, specific indicators, content flags, prototypical examples, and an overall score explanation.

While attempts to automate the scoring of IC exist (Conway et al., 2014; Houck et al., 2014), these are viewed as supplementary and not yet a substitute for human coding. However, they enhance our understanding of the complexity of the construct and highlight the need for more precise coding guidelines (Tetlock et al., 2014).

Another aspect to consider is whether IC is a stable trait of individuals or if it fluctuates based on environmental conditions, making it a state cognitive variable (Brodbeck et al., 2021). Current research has not addressed this question, and it is crucial to assess both profiles and procedures under varying uncertainty conditions within the decision-making paradigm.

In summary, this study identifies several knowledge gaps in the research on IC. These include IC’s relationships with other cognitive traits, the assessment methodologies employed in current research, the contemporary methods used to study IC, whether IC is a cognitive state or traits, and IC’s influence on the decision-making process.

To address these knowledge gaps, a systematic literature review was conducted using the AT framework, as exemplified in Tlili et al. (2022). AT has been utilized in various reviews to analyze different academic subjects, demonstrating its usefulness in conducting focused reviews centered on describing an activity rather than abstract theoretical concepts (e.g., Tlili et al., 2020; Sakallı et al., 2021).^1^ Viewing the research on IC as an activity with multiple significant components, the AT framework allows us to explore research as an activity interconnecting people, practices, and concepts in a complex task, providing insights into procedural and thematic topics related to IC and decision-making.^2^ In the AT framework, activity is seen as a system where objects and motives give meaning to the actions of agents, strongly articulated through mental intervention models in a problem situation developed by these agents in their professional work (Erausquin, 2014). AT enables us to conceptualize research as an activity involving multiple levels of mental models and human agents dealing with a specific problem.

Furthermore, neuroimaging, neuromodulation, and electrophysiology can reveal how the brain responds to different stimuli. By examining the underlying biochemical mechanisms involved in cognitive and physiological processes, we can better understand how IC operates^3^ and its significance in decision-making across various contexts.^4^ Neuroscience can help determine whether IC is subject to neuronal plasticity, age, or gender and how it adapts to changing environments. Studying the brain regions involved in IC can also shed light on its relationship with other cognitive phenomena.

Activity theory provides a framework for understanding and analyzing research practices by considering six elements: object, subject, rules, community, division of labor, and outcomes. These elements interact, and tensions and contradictions can arise within these networks. Through the application of AT, common topics, practices, and designs related to IC can be identified.^5^ In this article, AT is used to describe current research on IC within the context of decision-making, guided by two research questions:

RQ1: How has research on the decision-making process with IC perceived cognitive processes in the last decade?

RQ2: Does the fusion of IC and neuroscience provide a more precise analysis of the decision-making process compared to approaches without a neuroscientific perspective on cognition?

The subsequent sections of the article present the systematic review methodology based on PRISMA guidelines, summarize the principal findings regarding AT components in research on IC and neuroscience, and conclude with the main implications, limitations, and future directions for research on the state of IC and neuroscience in the decision-making process.

## 2. Method

**FIGURE 1:**
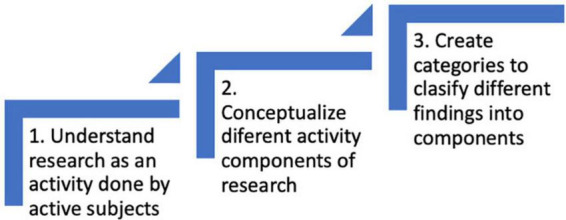
Steps for understanding research as an activity.

The study aims to utilize AT as a framework to analyze recent literature on IC, decision-making, and neuroscience. AT offers a comprehensive approach to analyzing any activity, including research or discourse analysis, by considering six interconnected elements. To employ this framework effectively, it is crucial to recognize that every activity serves a purpose and involves active subjects. Additionally, it is important to conceptualize the other components of the activity, such as the object, rules, community, division of labor, and outcomes. Finally, these conceptualizations create categories for classifying information (refer to Figure 1).

To conduct the study, the authors performed a content analysis of 31 relevant studies, identified using Atlas.ti. Subsequently, an additional search was conducted to identify 19 studies related to neuroscience and decision-making. The analysis included these studies to provide insights into the intersection of IC, decision-making, and neuroscience.

### 2.1. Screening procedures

The authors adhered to the PRISMA guidelines in conducting the literature review. Following the PICOS guidelines, they utilized three search strings in the EBSCO citation database. The inclusion and exclusion criteria were established and outlined in Table 1, which served as a reference for categorizing the articles based on analyzing their titles, abstracts, and content. The search focused exclusively on academic peer-reviewed articles published between 1 January 2012 and 31 December 2021.

**TABLE 1:** Search strategy for RQ1 following PICO guidelines (Jensen, 2022).

PICO element	Keywords	Search terms	Search strategies
Population	AT/decision making	Decision-making	Decision-making
Intervention	Integrative complexity	Integrative complexity	Integrative complexity OR Cognitive complexity
Comparison	Non-decision making/non-complexity	Decision and complexity without cognition	Complexity AND Decision NOT Integrative NOT Making
Outcome	Relevant features	Cognitive processes	Cognition OR cognitive function

Overall, the study aims to identify the most frequently mentioned concepts in literature falling within each of the components of an activity using AT. Using this approach, the authors hope to illuminate the relationship between IC, decision-making, and neuroscience.

**FIGURE 2:**
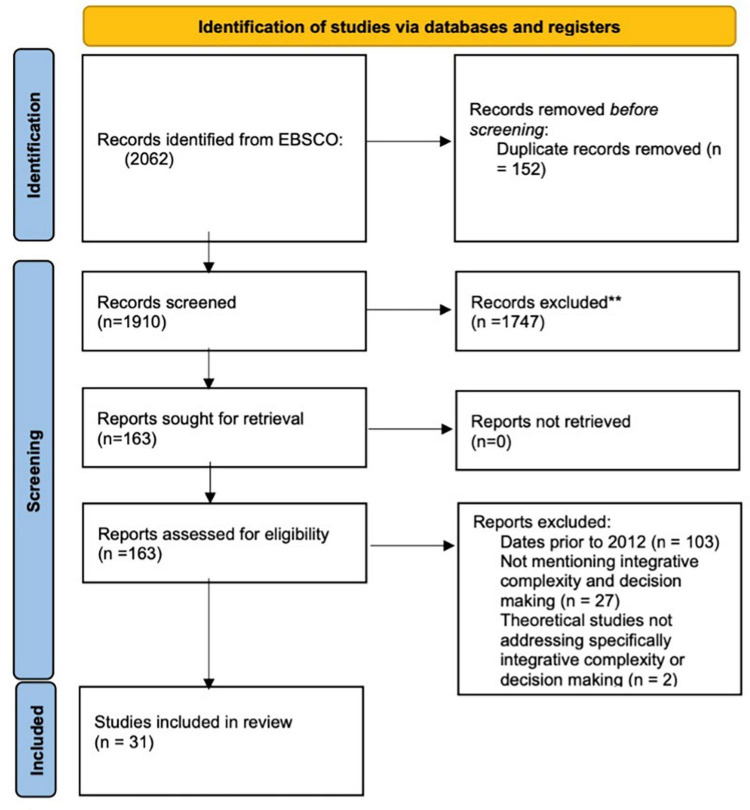
Flowchart for the article search and selection process using PRISMA guidelines. Adapted from Page et al. (2021). **The main criterion for exclusion was that a record was found to be unrelated to decision-making.

Following the selection process depicted in Figure 2, the initial database search produced 2,062 articles. After removing duplicates, 1,910 papers remained. Subsequently, through the screening process, 1,747 papers were excluded based on the established criteria. The inclusion criteria outlined in Table 1 were then reapplied during a full-text revision of the remaining 163 papers. As a result, 129 records were excluded due to their lack of relevance to decision-making. Ultimately, 31 papers met the eligibility criteria for this systematic review and were subjected to the conducted content analysis.

Since the content of this search did not yield relevant results pertinent to the neuroscience field, a second expanded search was carried out in the Google Scholar search engine. This subsequent search produced 19 additional articles, which were incorporated into the final list of articles under consideration. This resulted in 50 articles processed for this study. See PICOS Table 2 for search strings.

**TABLE 2:** Search strategy for RQ2 following PICO guidelines (Jensen, 2022).

PICO element	Keywords	Search terms	Search strategies
Population	AT/decision making	Decision-making	Decision-making
Intervention	Integrative complexity	Integrative complexity	Integrative complexity OR Cognitive complexity
Comparison	Non-decision making/non-complexity Cognitive process	Decision and complexity without cognition	Complexity AND Decision AND Cognitive process NOT Integrative NOT Making
Outcome	Neuroscience	Neuroscience research	Neuroscience

**FIGURE 3:**
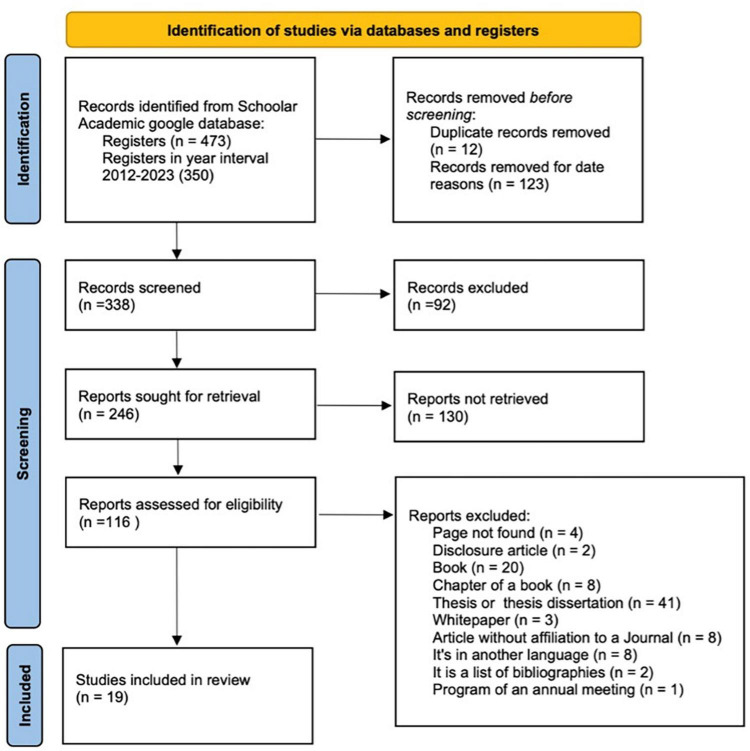
Flowchart for the article search and selection process using PRISMA guidelines in Google Scholar database. Adapted from Page et al. (2021).

In the search related to the field of neuroscience, a total of 473 results were obtained. After narrowing down the results to the last 10 years, 123 articles remained. Of these, 92 articles were excluded as they were not relevant to decision-making, IC, and neuroscience. Furthermore, 130 articles were not accessible, and 97 additional articles were excluded for various reasons. These included articles from pages that were not found (4), disclosure articles (2), books (20), book chapters (8), theses or dissertations (41), whitepapers (3), articles without affiliation to a journal (8), articles in languages other than English (8), bibliographies’ lists (2), or programs of annual meetings (1). Refer to Figure 3 for a visual representation of this process.

After the database search, articles were analyzed by title, abstract, and category. Categories were based on the inclusion and exclusion criteria defined in Table 3 for RQ1 and Table 4 for RQ2.

**TABLE 3:** Inclusion/exclusion criteria.

Inclusion criteria	Exclusion criteria
Empirical studies on integrative complexity and decision-making.	Theoretical studies do not address integrative complexity, neuroscience or decision-making.
Systematic reviews specifically addressing decision-making or integrative complexity.	Full text not available online.
Studies that are written in English.	Reports or white papers.
“Integrative complexity” and “decision-making” in keywords or references.	Other academic media (books, infographics, videos).
	Studies prior to 2012.

Elaborated according to PRISMA group guidelines (Moher et al., 2010).

**TABLE 4:** Inclusion/exclusion criteria.

Inclusion criteria	Exclusion criteria
Empirical studies on integrative complexity, decision-making, and neuroscience.	Theoretical studies do not address integrative complexity, neuroscience, or decision-making.
Systematic reviews specifically addressing decision-making, integrative complexity, and neuroscience.	Full text not available online, pages not found, and duplicates.
Studies that are written in English.	Studies that are written in another language.
“Integrative complexity,” “decision-making,” and “neuroscience” in keywords or references.	Other academic media (books, infographics, videos, chapters, disclosure article, thesis, and articles without affiliation to a journal).
	Studies prior to 2012.
	Reports or white papers.

Elaborated according to PRISMA group guidelines (Moher et al., 2010).

### 2.2. Conceptual framework

This study adopts the method proposed by Tlili et al. (2022) and applies AT (AT) and content analysis to analyze the 50 relevant studies identified using the PRISMA method. The research on IC, neuroscience, and decision-making is viewed as an activity seeking specific outcomes. According to AT, an activity is a system where objects and motives give meaning to actions (Erausquin, 2014). All components of human activity are interconnected.

The AT framework is an analytical tool recently utilized to summarize the literature on technology adoption in education (Sakallı et al., 2021; Tlili et al., 2022). It captures the components identified in the literature that moderate or mediate the relationship between IC, neuroscience, and decision-making. For instance, by applying AT, we can assess whether factors such as measurement, analytical methods, populations, or types of decisions studied can alter the effect of IC on decision-making outcomes.

**FIGURE 4:**
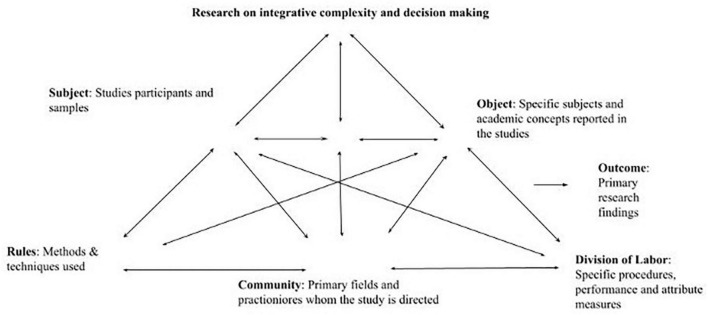
Activity theory framework with categories definition for content analysis of the present study. The arrows in the figure illustrate the interconnectedness of all components in an activity. Adapted from Tlili et al. (2020) and based on theoretical work on Engeström (2001).

Figure 4 illustrates the interaction between the different components of an activity. Just as every study adapts the definitions of these components to the specific activity of interest, our study follows suit. In the context of the research activity we are studying, the subject component refers to the participants or textual sources used in the initial 31 and subsequent 19 articles reviewed. The object component of the activity pertains to the constructs that the selected studies focused on. The rules component represents the methods employed in the studies.^6^ The community component refers to the audience or academic field to which the selected studies are directed. The division of labor component relates to the tools, data gathering, and data analysis employed in the research. Finally, the outcome component refers to the findings reported in both sets of articles reviewed.

The content analysis of the texts related to the activity of interest allows for identifying the most common features of each activity component. Figure 4 presents the layout of the AT components’ definitions and how they interact with each other in content analysis in this research.

### 2.3. Rigor and analysis

After establishing the categories within the AT framework, the final 31 and 19 articles were subjected to content analysis by two different researchers (refer to Supplementary material for complete database of articles, codes an categories). The content analysis was conducted in five stages, following the approach outlined by Kvale (2011) and utilizing the Atlas.ti software. The stages are described as follows:

Stage 1: The full-text articles were carefully examined, and relevant information related to research as an activity was condensed into brief synthetic statements that captured the essence of the main excerpts. Keywords were assigned to facilitate text identification.
Stage 2: The resulting statements and keywords were considered as quotes. The researchers familiarized themselves with these codes, compared their similarities and differences, and assessed their importance. The original text was referred to during this process to ensure the meaning was accurately preserved and understood.
Stage 3: The quotes’ frequencies, proximities, and context were further analyzed. They were then allocated to one of the six categories within the AT framework based on their relevance. If there were differences in the allocation of a quote by the researchers during this stage, they engaged in discussions to reconcile their different perspectives. They decided to relocate, rename, or remove the quote, all based on the previous stages of the analysis.
Stage 4: Once the quotes were allocated to categories, contingencies were examined to identify higher-level categories referred to as “codes.” These codes were used to relate the quotes to one another, the context, and the consequences of the action. The analysis of codes was based on discourse contingency, and only the most frequently occurring codes were established. Subsequently, these codes were merged into the components of AT as categories. Table 5 presents the final categories, codes, and quotes used for the analysis.

**TABLE 5:** Components, codes, and quotations used in content analysis.

Component	Codes for search on EBSCO database	Number of quotes for search on EBSCO database	Codes for search on Google Scholar database	Number of quotes for search on Google Scholar database
Subjects	Age and demographics Sample size Profession	31	Recorded text *Ad hoc* text Consumption patterns Patients	7
Objects	Disciplines Subdisciplines Interdisciplinary approach Specific topics	73	Politics Marketing Decision-making Neuroimaging Cortex Physiology Task and stimuli Cognition and psychological processes Other	110
Rules	Approach Procedures Methods	31	Content-coding texts Market analysis Case analysis Psychometric Experimental observation	5
Community	Academia Non-academia Mixed communities	31	Practitioners Neuroscience academics Decision scientist Multidisciplinary community Academics in a specific topic Cognitive and medical scholars	19
Division of labor	Techniques Instruments Analysis tools Online/face-to-face environment	31	Managing existing records Second order analytics Retrieving values of raw data Assessment or evaluations Expert survey	16
Outcomes	Main results of the study Considerations Theoretical insights Applied insights Disciplinary and interdisciplinary insights	31	Effectiveness of treatment Correlation between traits and neuroscience Prediction of behavior Correlation between traits and behavior Decision processes AI	18

Stage 5: Finally, the proximity, frequency, and context of the codes were established, and the description of the categories was developed.

The results obtained from the systematic reviews were analyzed separately and will be presented in the results section. These findings will then be integrated into the discussion section to compare the roles of neuroscience and IC in decision-making research, individually and in combination.

## 3. Results

The results will be presented in two subsections, each focusing on a different component of the AT framework. The first subsection, titled “Integrative Complexity Role,” will report the findings from the systematic review on the role of IC in decision-making, addressing RQ1. The second subsection, titled “Neuroscience Scope,” will address the role of neuroscience in decision-making, addressing RQ2.

### 3.1. Subjects

#### 3.1.1. Integrative complexity role

The “Subjects” component of the AT framework examines the characteristics of the research participants involved in the 31 reviewed studies. Based on the categories derived from the content analysis of the research papers, it was found that ten articles were literature reviews centered on critical and reflexive approaches. Additionally, 20 articles involved adult participants of both genders. One study reported a sample of 188 male teenagers with ADHD (Attention-Deficit/Hyperactivity Disorder) and 100 male teenagers without ADHD (Dekkers et al., 2020).

**FIGURE 5:**
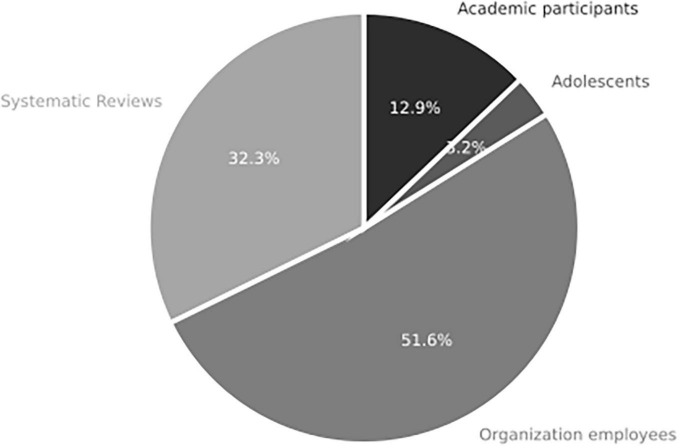
Sample type used in studies of systemic review addressing RQ1 on the role of integrative complexity in decision-making (*n* = 31 articles).

As depicted in Figure 5, the profile of the subjects in the reviewed studies varied, encompassing university communities, organizational environments, adolescents, and theoretical reviews. Of the 31 studies, 15 focused on the organizational environment and examined various levels, such as employees, decision-makers, executives, and work teams. One study specifically examined individuals close to former presidents of the USA and assessed how their personality traits influenced their decision-making processes (Gallagher and Allen, 2014). Another study collected data from a sample of 17,072 employees from Switzerland and Germany, roughly equivalent to Madison Square Garden seating capacity. This is an example of the power of sampling in subject research (Meynhardt et al., 2017). Of the studies examined, only four studies involved academic populations, three of which used student samples and one that involved both teachers and students. Notably, several studies utilized large sample sizes, with one study having a sample size of over 1,000 participants (Zhang et al., 2015).

The “Subjects” component is identified as one of the major strengths in research on IC and decision-making. The populations studied primarily come from organizations and universities, exhibiting different professions and ages. However, there is a notable absence of research examining national, ethnic, gender, personality, political orientation, or income differences. Furthermore, there is a lack of studies that compare group-level IC with individual-level IC. These gaps in the literature represent opportunities for future research to explore and consider these factors’ influence on IC and decision-making.

#### 3.1.2. Neuroscience scope

**FIGURE 6:**
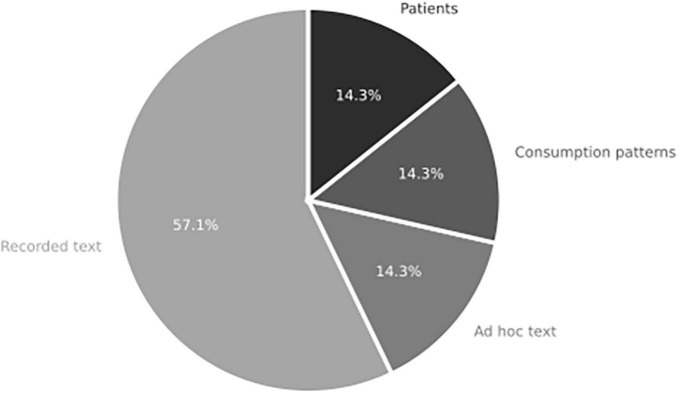
Subject component for studies in systemic review addressing RQ2 on the role of neuroscience and integrative complexity in decision-making (*n* = 19 articles).

When examining the intersection of neuroscience, decision-making, and IC, an interesting finding is that relatively few studies utilize actual data obtained from real samples. Out of the 19 studies reviewed, only five involved collecting primary data from actual subjects. However, even within these studies, the primary data often originated from other sources, such as text extracted from websites (Robinson et al., 2017), recorded texts from historical leaders (Rathbun, 2018; Arana, 2021), or consumption patterns derived from sales databases. Only two studies (Khan et al., 2013; Gola et al., 2015) collected data directly from experimental subjects, specifically medical patients (refer to Figure 6).

This finding highlights a limitation in the current research, as most studies rely on secondary data sources or simulated scenarios rather than gathering data directly from experimental subjects. The lack of studies with this kind of data presents an opportunity for future research to incorporate more studies that involve the collection of primary data, particularly from actual subjects in decision-making contexts.

### 3.2. Objects

#### 3.2.1. Integrative complexity role

The “Objects” component examines the topics covered in the reviewed articles. Only two studies (Zhang et al., 2015; Aleksovska, 2021) specifically utilize Suedfeld’s measure of IC as a variable of interest. However, all the articles address IC using alternative measures. For example, a computer science article (Silver, 2021) employs cognitive structure, which refers to the processes involved in dealing with information inputs (Ziv, 2011), as a measure of IC.

**FIGURE 7:**
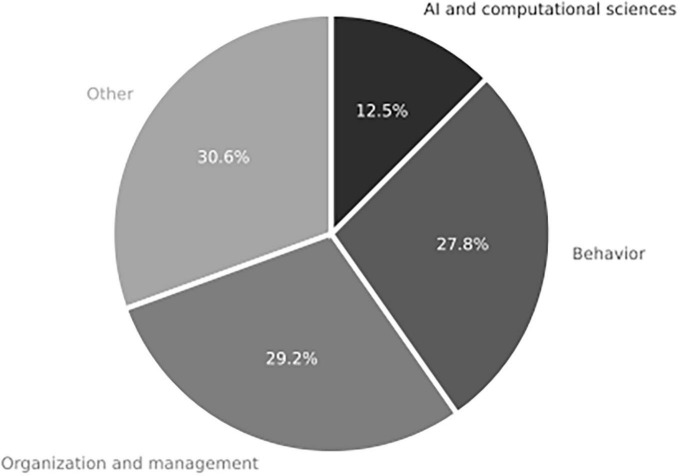
Object component in content analysis for systemic review addressing RQ1 on the role of integrative complexity in decision-making (*n* = 31 articles).

As shown in Figure 7, Decision-making in organizations is the most recurrent theme observed in these articles. This theme encompasses various topics, including abilities related to decision-making, leader performance, adequate conflict management, and corporate social responsibilities (Smith, 2014; Church et al., 2019; Selart et al., 2020).

The second most frequent topic encompasses themes related to behavioral sciences (20 quotes, 27.8%). These studies include topics such as cognitive structure and personality traits (Gallagher and Allen, 2014; Laureiro-Martinez and Brusoni, 2018; Dekkers et al., 2020).

The third most frequent topic in research on IC and decision-making pertains to computational sciences and artificial intelligence (AI) (9 quotes, 12.5%). One of the AI studies originates from computational sciences (Silver, 2021), while another is from health economics (Ogunbiyi et al., 2021).

Lastly, many articles covered a diverse range of topics that could not be classified into a single category (22 quotes, 30.6%). This “other” category includes articles on perception (Baba, 2018), cognition (Ziv, 2011), cognition and mindfulness (Selart et al., 2020), attitudes (Liu et al., 2015), emotion and emotional intelligence (Zhou et al., 2020), psychology and medicine (Dekkers et al., 2020), and personality (Gallagher and Allen, 2014; Tibon-Czopp et al., 2016). It also encompasses interdisciplinary studies that combine cognitive and management sciences (Meynhardt et al., 2017). Additionally, articles from disciplines such as sociology, anthropology, social psychology (Redd and Mintz, 2013; Carter and Philips, 2017), communications (Beck, 2019), philosophy (Wallis, 2015), and law (Moyer, 2012; Enns and Wohlfarth, 2013) are included within this category.

The frequencies observed indicate that the fields of organizational administration and management science have conducted more research on IC and decision-making than other fields. Numerous studies from these fields (Fern et al., 2012; Kownatzki et al., 2013; Thuan et al., 2016; Church et al., 2019; Neely et al., 2020) have contributed to understanding IC in decision-making. However, it is worth noting that there is significant multidisciplinary and interdisciplinary interest in this topic, with contributions from various fields.

Nevertheless, it is important to recognize that linking natural and social sciences in a single study is a broad approach. Most studies do not utilize Suedfeld’s measure of IC (Baker-Brown et al., 2008), which can lead to paradigmatic and ontological conflicts. These conflicts arise due to differences in the measures used to capture IC, such as whether they are general or relative, situation-oriented, or inherent to the individual, and so on.

To clarify and differentiate research lines, it is essential to distinguish between organizational IC and cognitive IC. For instance, Conway et al. (2008) distinguish between dialectical and elaborative IC. However, even these distinctions still utilize Suedfeld’s measure of IC as the basis for their analysis.

#### 3.2.2. Neuroscience scope

**FIGURE 8:**
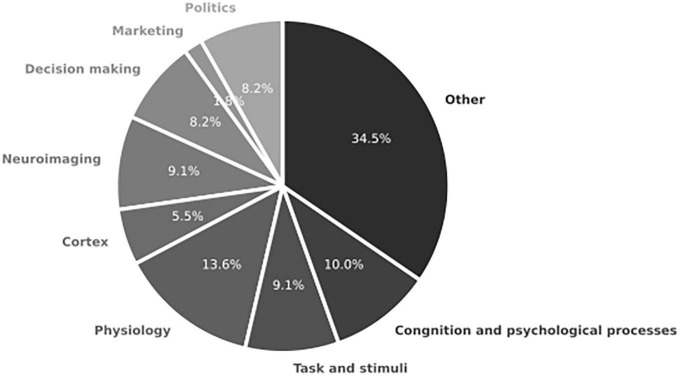
Object component for studies in systemic review addressing RQ2 on the role of neuroscience and integrative complexity in decision-making (*n* = 19 articles).

Expanding the search scope to include neuroscience in decision-making reveals limited direct attention given to IC (as shown in Figure 8). Instead, most of the Objects found in the review focus on physiological aspects of decision-making or mechanisms related to dealing with complexity. Studies such as those conducted by Starcke and Brand (2012), Summerfield and de Lange (2014), and Plassmann et al. (2015) explore the physiological aspects of the brain or stress mechanisms that influence the decision-making process. While these studies provide valuable insights into decision-making from a neuroscience perspective, they do not address IC directly.

In the broader scope of neuroscience research, researchers have focused on expanding stimuli to elicit neurological aspects of decision-making and IC. Tasks and stimuli range from gambling tests (Phelps et al., 2014) to chronometry tasks (Yeung and Summerfield, 2012), aiming to investigate the neural processes underlying decision-making and IC.

Neuroimaging techniques play a significant role in studying complexity and decision-making in neuroscience (13.7%). Functional magnetic resonance imaging (fMRI) is the primary instrument used for this purpose, as observed in studies by Phelps et al. (2014) and Plassmann et al. (2015). Other imaging devices, such as electroencephalography (EEG) (Kidd and Hayden, 2015), neural recordings (Summerfield and de Lange, 2014), and positron emission tomography (PET) (Starcke and Brand, 2012), are also employed. Within this category, there is a particular emphasis on exploring the brain cortex involved in decision-making, particularly the prefrontal area (Phelps et al., 2014; Summerfield and de Lange, 2014; Safron, 2020).

Cognitive and psychological processes (15.1%) are also highly interested in neuroscientific research on decision-making and IC. Topics such as curiosity (Kidd and Hayden, 2015), stress (Starcke and Brand, 2012), and personality (Arana, 2021) are explored within this domain. Furthermore, politics is another prominent object of study in neuroscience, particularly consumer behavior and political traits (Khan et al., 2013; Robinson et al., 2017). Decision-making itself is another area of great interest in neuroscientific research (12.3%), encompassing topics such as decision-making under risk (Starcke and Brand, 2012), decision-making styles (Connors et al., 2016), and social differences in decision-making (Yates and Oliveira, 2016).

The “other” category comprises a wide variety of specific Objects (34.5%) that do not fit into any other category. Examples include attraction (Coleman et al., 2017), AI (Safron, 2020), religiosity (Khan et al., 2013), storytelling (Gola et al., 2015), and others. It is important to note that most of these Objects are derived from academic reviews (14 out of 19), which provide interesting theoretical insights but lack empirical evidence-based support.

### 3.3. Rules

#### 3.3.1. Integrative complexity role

**FIGURE 9:**
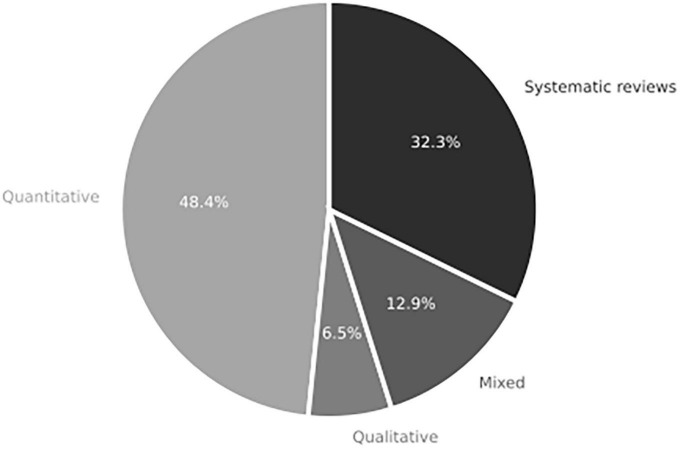
Distribution of methodological approach in studies in systemic review addressing RQ1 on the role of integrative complexity in decision-making (*n* = 31 articles).

The Rules component examines the methodological approaches employed in studying IC and decision-making. Content analysis identified four key methodological approaches: quantitative, qualitative, mixed methods, and systematic reviews. Figure 9 demonstrates that the quantitative approach is the most frequently observed, with 15 articles utilizing this method. Surveys and tests were commonly used in this approach, as seen in studies by Fern et al. (2012), Liu et al. (2015), Zhang et al. (2015), Meynhardt et al. (2017), Baba (2018), Church et al. (2019), Wang et al. (2019), and Zhou et al. (2020). One study conducted these techniques online (Behnke et al., 2020). Various analysis techniques were employed in the quantitative articles, including quantitative coding of documentary data (Foster and Keller, 2014), Chi-square hypothesis tests (Beck, 2019), and one-way ANOVA to identify differences between writing and among groups (Tibon-Czopp et al., 2016). Additionally, only three quantitative studies were experiments (Laureiro-Martinez and Brusoni, 2018; Dekkers et al., 2020; Aleksovska, 2021).

The least frequent methodological approach is mixed methods. Among the articles using this approach, two studies were identified integrating qualitative and quantitative techniques. For example, Kownatzki et al. (2013) integrate the use of grid analysis interviews with non-parametric factor analysis using custom-made t.o.p GRID software. In the same classification, Moyer (2012) performs a categoric analysis and logistic regression modeling.

Four studies use qualitative approaches. Two analyze interviews, documents, and observational data (Gallagher and Allen, 2014; Smith, 2014). The other two use content analysis of textual data, such as supreme court verdicts (Enns and Wohlfarth, 2013) or major decision-making models in foreign policy crises (Redd and Mintz, 2013).

The second most frequent methodological approach is systematic literature reviews (10 articles). Among the systematic reviews, literature reviews are the most common, with a total of six articles (Hahn and Aragon-Correa, 2015; Wallis, 2015; Thuan et al., 2016; Carter and Philips, 2017; Selart et al., 2020; Ogunbiyi et al., 2021). In addition, we found one metacritic study (Neely et al., 2020) and one report on empirical studies of AI-supported group decision-making (Silver, 2021).

The quantitative approach is predominant in studies that use surveys and questionnaires. These methods present a more effective use of time and resources and facilitate the study of opinions, attitudes, perceptions, and cognition. However, more complex methods may be appropriate when studying how emotions, social influence, or ambiguity influence decision-making processes (Bloemen et al., 2019).

The qualitative approach attempts to comprehend the complexity of reality taking into account variables from real-life outputs such as verdicts, international policy decisions, or surprise attacks. In comparison, quantitative approaches are more suited to develop abstract models of decision-making and deepen these for predicting individuals’ behavior. One strength of this approach is the amount of sample they gather and the control of different variables used in the studies. The connection of these approaches must be integrated regarding the main rules used to perform research and seen in an integrated approach rather than in an antagonistic role.

#### 3.3.2. Neuroscientific scope

As mentioned before, there is a clear predominance of academic reviews in this component (19/25). Only five studies report sampling and data analysis, among these, there are clearly both qualitative and quantitative methodological approaches. Robinson et al. (2017) uses text and video analysis to assess body movements and political orientations of several leaders and associate these patterns. In the same approach, Rathbun (2018) uses correspondence from Otto von Bismarck to draw conclusions of this leader for international policy and relates it to neuroscientific features.

The quantitative approach relies on psychometrics to assess the personality of leaders and their decision-making styles (Arana, 2021), and the only experiment found in the review assesses storytelling and social evaluation in patients with dementia (Gola et al., 2015). There is also market analysis that studies the pattern of consumption and political orientation regarding biases and information seeking (Khan et al., 2013; Jost, 2017).

### 3.4. Community

#### 3.4.1. Integrative complexity role

The community component examines the intended audience or academic field to which the selected studies are directed. The analysis of the 31 studies reveals that organizational administration and management science is the most prominent community in research on IC and decision-making. The studies within this field focus on topics such as leadership, conflict management, and corporate social responsibility (Thuan et al., 2016; Church et al., 2019; Neely et al., 2020).

Other communities contributing to IC and decision-making research include behavioral sciences, computational sciences, interdisciplinary studies, and various social sciences such as sociology, anthropology, and communication. These communities provide diverse perspectives and insights into understanding IC and decision-making (Redd and Mintz, 2013; Gallagher and Allen, 2014; Laureiro-Martinez and Brusoni, 2018; Beck, 2019).

While the community component of AT highlights the diverse range of fields and disciplines involved in research on IC and decision-making, there is a need for greater collaboration and integration across these communities. This would facilitate a more comprehensive and holistic understanding of the phenomenon and promote interdisciplinary dialogue and knowledge exchange.

Additionally, it is worth noting that there needs to be more representation from certain fields, such as neuroscience, in the reviewed studies. Despite the importance of neuroscience in understanding the neural mechanisms underlying decision-making and IC, its contribution to research in this area appears to be limited (Starcke and Brand, 2012; Summerfield and de Lange, 2014; Fonagy et al., 2015).

Fostering collaboration and knowledge exchange among different communities and disciplines can enrich the understanding of IC and decision-making, leading to more comprehensive and impactful research outcomes.

#### 3.4.2. Neuroscientific scope

**FIGURE 10:**
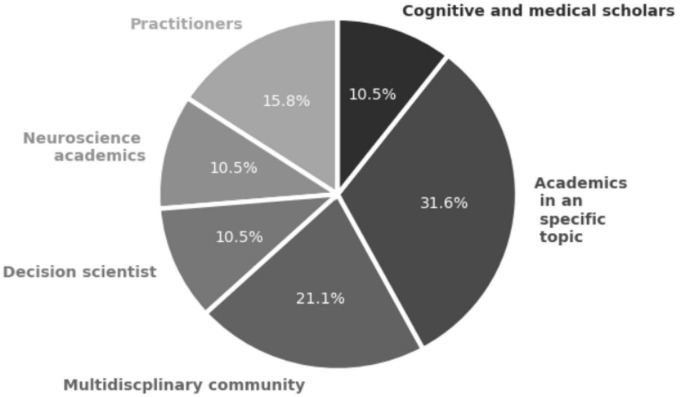
Community component for studies in systemic review addressing RQ2 on the role of neuroscience and integrative complexity in decision-making (*n* = 19 articles).

As mentioned earlier, academic reviews dominate the literature in this component, accounting for 19 out of 25 studies (see Figure 10). However, only five studies provide details on sampling and data analysis, employing both qualitative and quantitative methodological approaches.

Robinson et al. (2017) utilize text and video analysis to evaluate various leaders’ body movements and political orientations, establishing associations between these patterns. Similarly, Rathbun (2018) adopts a similar approach by analyzing correspondence from Otto von Bismarck to infer conclusions about his leadership in international policy, drawing connections to neuroscientific features.

On the other hand, the quantitative approach relies on psychometrics to assess leaders’ personality traits and their decision-making styles (Arana, 2021). Furthermore, the sole experiment identified in the review examines storytelling and social evaluation among patients with dementia (Gola et al., 2015).

In addition to these studies, a market analysis investigates consumption patterns and political orientations in relation to biases and information seeking (Khan et al., 2013).

### 3.5. Division of labor

#### 3.5.1. Integrative complexity role

The Division of Labor component focuses on the data collection procedures and measures employed in the 31 articles reviewed regarding IC and decision-making. These procedures are essential for validating research findings. For instance, in one study, six different evaluators were used to score test responses for assessing personality traits using the Rorschach test (Tibon-Czopp et al., 2016).

Measures of IC are also of interest in this component, as the reviewed studies utilized various measures. Through content analysis, three types of commonly used measures were identified. Suedfeld’s original measure of IC involves either human or AI scoring of textual statements. Despite the promotion of automated IC scoring tools over the past decade (Conway et al., 2014, 2018; Houck et al., 2014), these automated methods have not yet replaced human scoring. Consequently, most studies employ other variables that are simpler to implement as proxies for capturing IC.

The analysis of theoretical articles follows a meticulous six-step procedure, including selection, filtering, classification, back-and-forth scrutinizing, data extraction, and synthesis (Thuan et al., 2016). Temporality, spanning 20 years, is another valid research criterion for conducting systematic literature reviews (i.e., Carter and Philips, 2017).

Surveys are frequently utilized techniques in the reviewed studies. These survey-based studies establish reliability and validity in perceiving the decision-making process (Baba, 2018) or cognitive style (Meynhardt et al., 2017).

**FIGURE 11:**
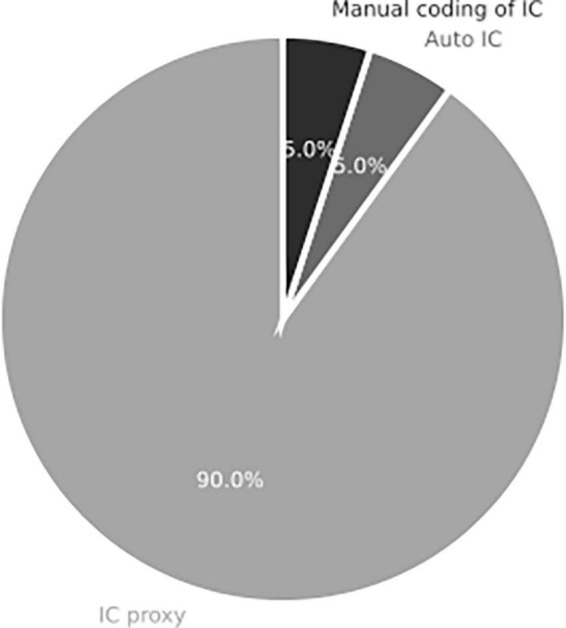
Measurement of IC for studies in systemic review addressing RQ1 on the role of integrative complexity in decision-making (*n* = 31 articles).

Regarding the measures used, Figure 11 illustrates that only two studies implemented Suedfeld’s original measure of IC. One study exclusively employed automated AI scoring (Aleksovska, 2021), while the other used the human-scored measure of IC (Zhang et al., 2015). The remaining 18 empirical studies reviewed used alternative variables as proxies for IC, such as cognitive complexity (Foster and Keller, 2014), decision-making strategies (Kownatzki et al., 2013), and discourse analysis of prime minister decisions (Ziv, 2011). Various tools for assessing IC exist, as manual scoring can be costly. However, automated scoring of IC has not yet been widely adopted. The resources and reliability required to assess IC have deterred researchers from directly assessing this feature, but it has not diminished the interest in the subject. Suedfeld’s measure of IC poses a research bottleneck that hinders its broader utilization. Nevertheless, the fact that most studies employed alternative variables as proxies for IC suggests that interest in the construct persists. In neuroscience, novel tools such as eye-tracking and fMRI are used to measure decision-making (Kim et al., 2012; Preuschoff et al., 2013).

Two studies in the reviewed literature employed online data collection procedures. One study conducted an online vignette experiment (Aleksovska, 2021), while the other utilized an online survey focusing on personality, cognition, and punishment (Behnke et al., 2020).

Most of the studies in the literature reviewed fall within the positivist paradigm. An example is an experimental study on corporate decision-making outcomes using students as corporation managers (Church et al., 2019). However, three studies deviate from the positivist paradigm regarding methods, focusing more on the context and relativity of data rather than the factual realization of the phenomenon. One study employs interviews (Smith, 2014), while another employs discourse analysis (Ziv, 2011). Both studies analyze the cognitive complexity of Israel’s former prime minister.

Research in the social sciences often incorporates descriptive and inferential statistics. The reviewed literature includes studies utilizing scale measures and specialized software, such as “t.o.p GRID,” which employed non-parametric factor analysis to interpret verbal data from interviews (Kownatzki et al., 2013).

The Division of Labor component highlights the various procedures and measures employed in the literature on IC and decision-making. The predominance of the positivist paradigm contributes to the factualization of the relationships between IC and decision-making by providing data gathering without the need for context interpretation or researcher bias. However, there is a need for more qualitative research on IC and decision-making to enhance ecological validity and the applicability of findings. Additionally, further measurement studies that assess the validity of AI-scored IC and compare it to simpler alternative measures would be beneficial in clarifying which measures better capture the construct and the trade-offs among different measurement methods.

#### 3.5.2. Neuroscience scope

**FIGURE 12:**
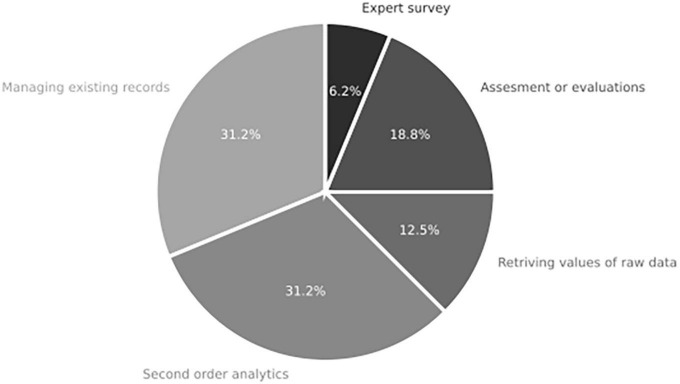
Division of labor component for studies in systemic review addressing RQ2 on the role of neuroscience and integrative complexity in decision-making (*n* = 19 articles).

The analysis of techniques and data processing in the reviewed literature (refer to Figure 12) reveals that various approaches are employed. Some studies focus on managing existing records, such as sorting websites or sampling texts, as seen in Robinson et al. (2017). However, there is a predominant trend toward conducting second-order analytics to extract elaborated values from other sources. For instance, studies retrieve data from sales databases (Khan et al., 2013) or analyze posts collected from webpages (Arana, 2021).

Additionally, some studies directly involve participants in the analysis process. Assessments of social interactions (Arana, 2021) or the application of psychometrics to measure subjects’ characteristics (Gola et al., 2015).

### 3.6. Outcomes

#### 3.6.1. Integrative complexity role

Several studies define decision-making as a cognitive capacity, ability, or set of strategies. Regarding decision-making processes, cognitive traits are often considered more important than any specific cognitive style.^7^ For instance, Zhang et al. (2015) find a positive correlation between holistic thinking, IC, and competent, adaptable, and proactive management decisions made by supervisors regarding their subordinates. This finding helps explain why managers tend to be cautious about implementing radical changes in uncertain and complex conditions. Experimental evidence has supported the positive correlation between IC and decision-making performance (Laureiro-Martinez and Brusoni, 2018), and survey studies have also corroborated this relationship (Meynhardt et al., 2017; Baba, 2018; Wang et al., 2019; Dekkers et al., 2020; Aleksovska, 2021). Additionally, studies have found that emotions modulate individuals’ confidence in making good decisions in corporate and clinical settings (Selart et al., 2020; Zhou et al., 2020).

**FIGURE 13:**
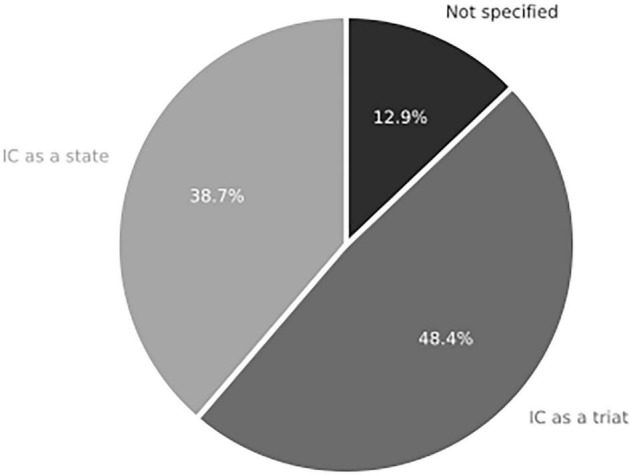
Definition of IC as a state or trait in systemic review addressing RQ1 on the role of integrative complexity in decision-making (*n* = 31 articles).

The content analysis of the Outcomes component in Figure 13 indicates significant debate regarding whether IC should be considered a state or trait. Among the analyzed articles, 15 assume it to be a state, while 12 (48.4%) treat it as a cognitive trait. Only four studies refrain from definitively categorizing IC as one or the other. Over the past decade, research has not provided a clear consensus on whether IC is a stable personality trait or a cognitive state variable that can be influenced by environmental conditions (Brodbeck et al., 2021). It is important to note that defining IC as a state or trait can have different implications for decision-making processes and outcomes.

One important consideration is whether there should be a push for more consistent research standards. Researchers argue that more systematic approaches are needed to understand how cognition, values, and perceptions influence executive strategies and improve organizational performance (Neely et al., 2020). Smith (2014) also highlights that IC is related to managing strategic paradoxes and management dilemmas, emphasizing the need for further exploration.

In summary, the analysis of the Outcomes component reveals that IC and decision-making are interconnected across various settings, ranging from organizational to clinical environments. However, IC is often intertwined with other cognitive processes, such as intelligence and attention, making it challenging to precisely estimate the strength of the relationship between IC and decision-making.

Furthermore, decision-making variables have predominantly focused on the individual, while factors that impact group decision-making, such as gender, ethnicity, social interaction, and affect, are often overlooked. Finally, distinguishing types of IC by field, such as political versus organizational IC, may provide greater clarity on disciplinary trends in IC and decision-making.

#### 3.6.2. Neuroscience scope

The analysis of Outcomes in the research indicates a notable interest in exploring the relationship between traits and neuroscience. Studies aim to investigate complex motivational functions supported by the lateral prefrontal cortex (Dixon et al., 2017) and draw inferences beyond simple brain-behavior correlations (Plassmann et al., 2015). Furthermore, some Outcomes attempt to establish correlations between traits and behavior, such as automatic response and learning (Starcke and Brand, 2012) or task and motivation (Kidd and Hayden, 2015).

The implications of Outcomes for the decision-making process are also evident in the research. Findings related to decision-making include the exploration of possibilities, trade-offs, and implementation (Yates and Oliveira, 2016). The deconstruction of the decision-making process into benefits, opportunities, costs, and risks is also mentioned (Saaty, 2015). Moreover, there is a recognition of the wide variety of individual differences in this subject across people (Connors et al., 2016).

## 4. Conclusion, limitations, and future directions

This study presents a systematic literature review of 31 recent studies that analyze the relationship between IC and decision-making. The analysis was conducted using content analysis and the AT framework to assess major research components as an activity. The study identified key trends, gaps, and challenges in IC and decision-making.

The results indicate the need for a more specific definition of IC to assess its relationship with decision-making. The distinction between IC as a state or trait variable still needs to be solved, and manual and automated coding techniques lack differentiation. The measurement of IC remains costly and time-consuming, posing operational barriers for research in this field.

Using the AT framework allowed for a systematic assessment of the research network and revealed the impact of changes in IC measurement on research themes, approaches, participants, and outcomes. The review also highlights the need for a diverse community of researchers to address the products and challenges in this field.

Expanding the search to the neuroscience scope identified the need for more experimental efforts and controlled settings to test theoretical findings directly. The outcomes of neuroscience research show promise in assessing physiological and behavioral processes related to complexity but require a focused line of research for greater clarity and sharing of findings. The methods and techniques employed in this field are diverse, reflecting the complexity of research on IC and decision-making.

The study identifies two knowledge gaps for future research. The first is the measurement ambiguity of IC, which requires further validation and accessibility studies of automated scoring programs. It is also crucial to differentiate IC from other cognitive features and determine its trait or state nature. The second gap is the lack of detailed information on procedures, models, and tools used in research on IC and decision-making. Addressing these gaps will contribute to advancing the field and understanding the relationship between IC and decision-making.

In conclusion, the study highlights important limitations, suggestions, and conclusions regarding IC and decision-making. The identified gaps provide directions for future research, including examining individual differences in IC, establishing experimental standards, and integrating findings from neuroscience. A summarized overview of these limitations, suggestions, and conclusions can be found in Table 6.

**TABLE 6:** Synthesis of limitations, suggestions, and conclusions from the systematic reviews.

Components	Gaps addressed	Limitations	Suggestions	Conclusions
Subjects	(b) How IC is been assessed in current research and (c) what are the current methods to study IC.	Minor experimental data, population of experimental studies mainly from academia and organizational environments.	Extend the studies to more populations outside the academia and organizations and focus on more sociodemographic traits (gender, ethnicity, and among others). Perform more experimental studies besides systematic reviews.	This component is a major strength for study IC in the decision-making process, but it needs to be broadened.
Objects	(a) The relationship of IC with other cognitive traits, (b) how IC is been assessed in current research, (c) what are the current methods to study IC, (d) is IC a cognitive state or trait, and (e) what is the influence of IC on the decision-making process.	IC asses mainly with proxy variables, IC influence on decision-making is considered but not specified.	Specify the role of IC in studies, use Suedfeld’s measure on IC.	There is a great diversity of objects and topics related to IC in the decision-making process, but this relation needs to be more concise and integrated with theory.
Rules	(a) The relationship of IC with other cognitive traits, (c) what are the current methods to study IC, and (e) what is the influence of IC on the decision-making process.	There is a predominance of systematic reviews in the comprehension of IC, but it is not integrated with major gaps in literature.	Conduct more experimental research, link findings with major gaps in literature.	There exists a diversity of studies directed to the comprehension of IC, but these approaches need to be integrated with major gaps in literature on the concept.
Community	(a) The relationship of IC with other cognitive traits, (b) how IC is been assessed in current research, (c) what are the current methods to study IC, (d) is IC a cognitive state or trait. and (e) what is the influence of IC on the decision-making process.	There is a main focus on academic audiences.	The study of IC should be more practical in reaching wider audiences, the neuroscientific scope seems to be particularly useful in this question.	There has been a major interest on an academic audience on the study of IC in the decision-making process, nonetheless, there seem to be an opening in directing these efforts to other communities.
Division of labor	(b) How IC is been assessed in current research and (c) what are the current methods to study IC.	Few studies code IC directly (manual or automatic).	There should be an emphasis on measuring IC directly without any proxies.	A better automatic tool to assess IC could foster further experimental research that considers IC without any proxies.
Outcomes	(d) Is IC a cognitive state or trait and (e) what is the influence of IC on the decision-making process.	There have been little attentions to the cognitive features of IC in the studies, and there are few articles that specify IC as a state or trait.	Specify IC in terms of their cognitive features, create more consistent standards on IC research.	There is a great diversity of variables associated with IC and decision-making in terms of performance, styles, and tradeoffs. Also, there is an interest of the physiological structures and processes related to IC in decision-making.

Addressing these knowledge gaps can allow for systematic comparison of experimental results across fields and applications and further our understanding of how IC impacts decision-making outcomes in different decision contexts.

## Data availability statement

The original contributions presented in this study are included in the article/Supplementary material, further inquiries can be directed to the corresponding author.

## Author contributions

IM, EM-P, and MT-R designed the research, analyzed the results, and wrote the manuscript. HZ-M, LS-B, and AD collaborated with reviewing, analyzing, and discussing the reviewed literature. YO and VE-J cleaned the databases and helped with statistical analysis. All authors contributed to the article and approved the submitted version.

## Footnotes

1 Activity theory is based on Vygotsky and Cole’s (1978) work on mediated activity. According to the author, every activity is not just the result of internal motivation or an isolated impulse. Instead, all motives for action are filtered and shaped by various aspects of the environment, which can be material or symbolic and are referred to as “tools” because they enable the production of an outcome (as shown in Figure 10). In this article, we view research as a mediated activity, where different elements interact to achieve a specific outcome. These elements, as proposed by Tlili et al. (2022), provide categories for systematic reviews, thereby enhancing the conceptual framework of the information reviewed.

2 AT framework described by the authors in which this article is based is used to perform content analysis on the interplay of various components and actors involved in research. Since activity is defined as a system of purposeful behaviors leading to recognizable changes in human practices (Kim et al., 2012).

3 In sum integrative complexity’s relation to other cognitive characteristics of the person remains unclear, but it is evident its role in the outcome of decisions e.g., political, medical, and managerial.

4 So, we propose that IC could be a mediating tool in decision-making between the decision maker and the outcome of such decision.

5 AT framework is useful on understanding the mediating role of integrative complexity in the decision-making process.

6 Due to the mediating nature of subject, object, and tools, some specific terms may be conflated into distinct categories. For instance, FMRI can serve as a tool if it is only used to retrieve data, as part of a study design if it is compared to another imaging technique, or as a method if researchers report it as such. For this systematic review, the coders followed the part description and assigned each term to the category that best suited its description in each paper.

7 For cognitive style we refer to the combination of cognitive states and traits that a person exhibits during and specific situation, this is highly oriented to external stimulus and although it has stability can be modulated by external factors.

## References

[B1] Aleksovska M. (2021). Accountable for what? The effect of accountability standard specification on decision-making behavior in the public sector. *Public Perform. Manag. Rev.* 44 707–735. 10.1080/15309576.2021.1900880

[B2] Arana I. (2021). The personalities of presidents as independent variables. *Polit. Psychol.* 42 695–712. 10.1111/pops.12722

[B3] Baba M. M. (2018). Decision making and demographics: A study of academicians in Indian universities. *J. Organ. Hum. Behav.* 7 23–44.

[B4] Baker-Brown G. Ballard E. J. Block S. de Vries B. de Vries P. Suedfeld P. (2008). “Coding manual for conceptual/integrative complexity,” in *Motivation and personality: Handbook of thematic content analysis*, eds Smith C. P. Atkinson J. W. McClelland D. C. Veroff J. (Cambridge: Cambridge University Press), 401–418. 10.1017/CBO9780511527937

[B5] Beck S. J. (2019). Majority and minority subgroup argumentation messages. *Commun. Rep.* 32 69–82. 10.1080/08934215.2019.1603317

[B6] Behnke A. Strobel A. Armbruster D. (2020). When the killing has been done: Exploring associations of personality with third-party judgment and punishment of homicides in moral dilemma scenarios. *PLoS One* 15:e0235253. 10.1371/journal.pone.0235253 32603338 PMC7326181

[B7] Békes V. Suedfeld P. (2019). “Integrative complexity,” in *Encyclopedia of personality and individual differences*, eds Zeigler-Hill V. Schackelford T. (Cham: Springer).

[B8] Bloemen P. J. T. M. Marchau V. A. W. J. Popper S. W. Walker W. E. (eds) (2019). *Decision making under deep uncertainty: From theory to practice.* Cham: Springer International Publishing.

[B9] Brodbeck F. C. Kugler K. G. Fischer J. A. Heinze J. Fischer D. (2021). Group-level integrative complexity: Enhancing differentiation and integration in group decision-making. *Group Process. Intergr. Relat.* 24 125–144. 10.1177/1368430219892698

[B10] Carter A. B. Philips K. W. (2017). The double-edged sword of diversity: Toward a dual pathway model. *Soc. Pers. Psychol. Compass* 11:e12313. 10.1111/spc3.12313

[B11] Church B. K. Jiang W. Kuang X. Vitalis A. (2019). A dollar for a tree or a tree for a dollar? The behavioral effects of measurement basis on managers’ CSR investment decision. *Account. Rev.* 94 117–138. 10.2308/accr-52332

[B12] Coleman P. Coon D. Kim R. Chung C. Bass R. Regan B. (2017). Promoting constructive multicultural attractors: Fostering unity and fairness from diversity and conflict. *J. Appl. Behav. Sci.* 53 180–211. 10.1177/0021886317702133

[B13] Connors B. Rende R. Colton T. (2016). Beyond self-report: Emerging methods for capturing individual differences in decision-making process. *Front. Psychol.* 7:312. 10.3389/fpsyg.2016.00312 26973589 PMC4776304

[B14] Conway L. G. Suedfeld P. Tetlock P. E. (2018). “Integrative complexity in politics,” in *The Oxford handbook of behavioral political science*, eds Mintz A. Terris L. G.. 10.1093/OXFORDHB/9780190634131.013.7

[B15] Conway L. G. Woodward S. R. (2019). Integrative complexity across domains and across time: Evidence from political and health domains. *Pers. Individ. Differ.* 155:109713. 10.1016/j.paid.2019.109713

[B16] Conway L. G. Conway K. R. Gornick L. J. Houck S. C. (2014). Automated integrative complexity. *Polit. Psychol.* 35 603–624. 10.1111/pops.12021

[B17] Conway L. G. Thoemmes F. Allison A. M. Towgood K. H. Wagner M. J. Davey K. (2008). Two ways to be complex and why they matter: Implications for attitude strength and lying. *J. Pers. Soc. Psychol.* 95:1029. 10.1037/a0013336 18954192

[B18] Dekkers T. J. Huizenga H. M. Popma A. Bexkens A. Zaleedar J. N. Jansen B. R. J. (2020). Decision-making deficits in adolescent boys with and without Attention-Deficit/Hyperactivity Disorder (ADHD): An experimental assessment of associated mechanisms. *J. Abnorm. Child Psychol.* 48 495–411. 10.1007/s10802-019-00613-7 31883040

[B19] Dixon M. L. Thiruchselvam R. Todd R. Christoff K. (2017). Emotion and the prefrontal cortex: An integrative review. *Psychol. Bull.* 143:1033–1081. 10.1037/bul0000096. 28616997

[B20] Driver M. J. Streufert S. (1969). Integrative complexity: An approach to individuals and groups as information-processing systems. *Administr. Sci. Q.* 14 272–285. 10.2307/2391105

[B21] Engeström Y. (2001). Expansive learning at work: Toward an activity theoretical reconceptualization. *J. Educ. Work* 14 133–156. 10.1080/13639080020028747

[B22] Enns P. K. Wohlfarth P. C. (2013). The Swing Justice. *Journal of Politics* 75 1089–1108.

[B23] Erausquin C. (2014). La teoría histórico-cultural de la actividad como artefacto mediador para construir intervenciones en indagaciones sobre el trabajo de psicologos en escenarios educatiuvos. *Rev. Segunda Epoca* 13 172–197.

[B24] Fern M. J. Cardinal L. B. O’Neill H. M. (2012). The genesis of strategy in new ventures: Escaping the constraints of founder and team knowledge. *Strateg. Manag. J.* 33 427–448. 10.1002/smj.1944

[B25] Fonagy P. Luyten P. Bateman A. (2015). Translation: Mentalizing as treatment target in borderline, personality disorders. *Pers. Disord. Theory Res. Treat.* 6 380–392. 10.1037/per0000113 26436581

[B26] Foster D. M. Keller J. W. (2014). Leaders’ cognitive complexity, distrust, and the diversionary use of force. *Foreign Policy Anal.* 10 205–224. 10.1111/fpa.12019

[B27] Gallagher M. E. Allen S. H. (2014). Presidential personality: Not just a nuisance. *Foreign Policy Anal.* 10 1–22. 10.1111/fpa.12006

[B28] Gola K. A. Thorne A. Veldhuisen L. D. Felix C. M. Hankinson S. Pham J. (2015). Neural substrates of spontaneous narrative production in focal neurodegenerative disease. *Neuropsychologia* 79, 158–171. 10.1016/J.NEUROPSYCHOLOGIA.2015.10.022 26485159 PMC4809527

[B29] Hahn T. Aragon-Correa A. J. (2015). Toward cognitive plurality on corporate sustainability in organizations: The role of organizational factors. *Organ. Environ.* 28 255–263. 10.1177/1086026615604446

[B30] Houck S. C. Conway L. G. Gornick L. J. (2014). Automated integrative complexity: Current challenges and future directions. *Polit. Psychol.* 35 647–659. 10.1111/pops.12209

[B31] Jensen K. (2022). *Evidence-based nursing practice: 7 steps to the perfect PICO search what is evidence-based nursing practice and why is it important?* In CINAHL. EBSCO Health, 1–9. Available online at: https://www.ebsco.com/sites/g/files/nabnos191/files/acquiadam-assets/7-Steps-to-the-Perfect-PICO-Search-White-Paper_0.pdf

[B32] Jost J. (2017). The marketplace of ideology: “Elective affinities” in political psychology and their implications for consumer behavior. *J. Consum. Psychol.* 27 502–520. 10.1016/j.jcps.2017.07.003

[B33] Khan R. Misra K. Singh V. (2013). Ideology and brand consumption. *Psychol. Sci.* 24 326–333. 10.1177/0956797612457379 23381562

[B34] Kidd C. Hayden B. (2015). The psychology and neuroscience of curiosity. *Neuron Perspect.* 88 449–460. 10.1016/j.neuron.2015.09.010 26539887 PMC4635443

[B35] Kim B. E. Seligman D. Kable J. W. (2012). Preference reversals in decision making under risk are accompanied by changes in attention to different attributes. *Front. Neurosci*. 6:109. 10.3389/fnins.2012.00109 22833715 PMC3400145

[B36] Kownatzki M. Walter J. Floyd S. W. Lechner C. (2013). Corporate control and the speed of strategic business unit decision making. *Acad. Manag. J.* 56 1295–1325. 10.5465/amj.2011.0804

[B37] Kvale S. (2011). in *Las entrevistas en investigación cualitativa*, Trans Blanco Castellano C. del Amo Martín T. (Madrid: Ediciones Morata, S.L).

[B38] Laureiro-Martinez D. Brusoni S. (2018). Cognitive flexibility and adaptive decision-making: Evidence from a laboratory study of expert decision makers. *Strateg. Manag. J.* 39 1031–1058. 10.1002/smj.2774

[B39] Liu Y.-L. Keeling K. A. Papamichail N. K. (2015). Should retail trade companies avoid recruiting maximisers? *Manag. Decis.* 53 730–751. 10.1108/MD-06-2014-0402

[B40] McCullough H. (2019). Be complex, be very complex: Evaluating the integrative complexity of main characters in horror films. *Psychol. Popular Media Cult.* 10 50–58. 10.1037/ppm0000266

[B41] Meynhardt T. Hermann C. Anderer S. (2017). Making sense of a most popular metaphor in management: Towards a HedgeFox scale for cognitive styles. *Administr. Sci.* 7 33–56. 10.3390/admsci7030033

[B42] Moher D. Liberati A. Tetzlaff J. Altman D. G. PRISMA Group (2010). Preferred reporting items for systematic reviews and meta-analyses: The PRISMA statement. *Int. J. Surg.* 8 336–341. 10.1016/j.ijsu.2010.02.007 20171303

[B43] Moyer L. P. (2012). The role of case complexity in judicial decision making. *Law Policy* 34 291–313. 10.1111/j.1467-9930.2012.00362.x

[B44] Neely B. H. Lovelace J. B. Cowen A. P. Hiller N. J. (2020). Metacritiques of upper echelons theory: Verdicts and recommendations for future research. *J. Manag.* 46 1029–1063. 10.1177/0149206320908640

[B45] Ogunbiyi N. Basukoski A. Chaussalet T. Batarseh F. A. (2021). An exploration of ethical decision making with intelligence augmentation. *Soc. Sci.* 10 57–71. 10.3390/socsci10020057

[B46] Page M. McKenzie J. Bossuyt P. Boutron I. Hoffmann T. Mulrow C. (2021). The PRISMA 2020 statement: An updated guideline for reporting systematic reviews. *BMJ* 372:n71. 10.1136/bmj.n71 33782057 PMC8005924

[B47] Peterson R. S. Owens P. D. Tetlock P. E. Fan E. T. Martorana P. (1998). Group dynamics in top management teams: Groupthink, vigilance, and alternative models of organizational failure and success. *Organ. Behav. Hum. Decis. Process.* 73 272–305. 10.1006/obhd.1998.2763 9705805

[B48] Phelps E. Lempert K. Sokol-Hessner P. (2014). Emotion and decision making: Multiple modulatory neural circuits. *Annu. Rev. Neurosci.* 37 263–287. 10.1146/annurev-neuro-071013-014119 24905597

[B49] Plassmann H. Venkatraman V. Huettel S. Yoon C. (2015). Consumer neuroscience: Applications, challenges, and possible solutions. *J. Market. Res.* 52 427–435. 10.1509/jmr.14.0048 11670861

[B50] Preuschoff K. Mohr P. N. C. Hsu M. (2013). Decision making under uncertainty. *Front. Neurosci.* 7:218. 10.3389/FNINS.2013.00218/BIBTE PMC3834552 24311997

[B51] Rathbun B. (2018). The rarity of realpolitik what Bismarck’s rationality reveals about international politics. *Int. Secur.* 43 7–55. 10.1162/ISEC_a_00323

[B52] Redd S. B. Mintz A. (2013). Policy perspectives on national security and foreign policy decision making. *Policy Stud. J.* 41 s11–s37. 10.1111/psj.12010

[B53] Robinson M. Boyd R. L. Fetterman A. Michelle R. (2017). The mind versus the body in political (and nonpolitical) discourse: Linguistic evidence for an ideological signature in U.S. politics. *J. Lang. Soc. Psychol.* 36 438–461. 10.1177/0261927X16668376

[B54] Saaty T. (2015). The modern science of multicriteria decision making and its practical applications: The AHP/ANP approach. *Operat. Res.* 61 1101–1118. 10.1287/opre.2013.1197 19642375

[B55] Safron A. (2020). An Integrated World Modeling Theory (IWMT) of consciousness: Combining integrated information and global neuronal workspace theories with the free energy principle and active inference framework; toward solving the hard problem and characterizing agentic causation. *Front. Artif. Intell.* 3:330. 10.3389/frai.2020.00030 33733149 PMC7861340

[B56] Sakallı Ö Tlili A. Altınay F. Karaatmaca C. Altınay Z. Dağı G. (2021). The role of tolerance education in diversity management: A cultural historical activity theory perspective. *SAGE Open* 11 1–11. 10.1177/21582440211060831

[B57] Selart M. Schei V. Lines R. Nesse S. (2020). Can mindfulness be helpful in team decision-making? A framework for understanding how to mitigate false consensus. *Eur. Manag. Rev.* 17 1015–1027. 10.1111/emre.12415

[B58] Shao Y. Nijstad B. A. Täuber S. (2019). Creativity under workload pressure and integrative complexity: The double-edged sword of paradoxical leadership. *Organ. Behav. Hum. Decis. Process.* 155 7–19. 10.1016/j.obhdp.2019.01.008

[B59] Silver S. D. (2021). Dynamics of Negative evaluations in the information exchange of interactive decision-making teams: Advancing the design of technology-augmented GDSS. *Inform. Syst. Front.* 23 1621–1643. 10.1007/s10796-020-10063-y

[B60] Smith W. (2014). Dynamic decision making: A model of senior leaders managing strategic paradoxes. *Acad. Manag. J.* 57 1592–1624. 10.5465/amj.2011.0932

[B61] Starcke K. Brand M. (2012). Decision making under stress: A selective review. *Neurosci. Biobehav. Rev.* 36 1228–1248. 10.1016/j.neubiorev.2012.02.003 22342781

[B62] Suedfeld P. (2010). The cognitive processing of politics and politicians: Archival studies of conceptual and integrative complexity. *J. Pers.* 78 1668–1702. 10.1111/j.1467-6494.2010.00666.x 21039528

[B63] Suedfeld P. Bluck S. (1988). Changes in integrative complexity prior to surprise attacks. *J. Conflict Resolut.* 32:626.

[B64] Suedfeld P. Guttieri K. Tetlock P. E. (2010). “Assessing integrative complexity at a distance: Archival analyses of thinking and decision making,” in *The psychological assessment of political leaders: with profiles of Saddam Hussein and Bill Clinton*, ed. Post J. D. (University of Michigan Press), 246–270.

[B65] Suedfeld P. Tetlock P. E. Streufert S. (1992). “Conceptual/integrative complexity,” in *Handbook of thematic content analysis*, ed. Smith C. P. (Cambridge: Cambridge University Press), 393–400. 10.1017/CBO9780511527937.028

[B66] Summerfield C. de Lange F. (2014). Expectation in perceptual decision making: Neural and computational mechanisms. *Nat. Rev. Neurosci.* 15 745–56. 10.1038/nrn3838 25315388

[B67] Tetlock P. E. (1986). A value pluralism model of ideological reasoning. *J. Pers. Soc. Psychol.* 50 819–827. 10.1037/0022-3514.50.4.819

[B68] Tetlock P. E. Metz S. E. Scott S. E. Suedfeld P. (2014). Integrative complexity coding raises integratively complex issues. *Polit. Psychol.* 35 625–634.

[B69] Tetlock P. E. Peterson R. S. Berry J. M. (1993). Flattering and unflattering personality portraits of integratively simple and complex managers. *J. Pers. Soc. Psychol.* 64 500–511. 10.1037/0022-3514.64.3.500

[B70] Thuan N. Antunes P. Johnstone D. (2016). Factors influencing the decision to crowdsource: A systematic literature review. *Inform. Syst. Front.* 18 47–69. 10.1007/s10796-015-9578-x

[B71] Tibon-Czopp S. Appel L. Zeligman R. (2016). Assessing personality patterns of functioning in a decision-making ambiguous task: The Rorschach Reality-Fantasy Scale (RFS-2). *Group Decis. Negotiation* 25 65–76. 10.1007/s10726-015-9432-z

[B72] Tlili A. Denden M. Duan A. Padilla-Zea N. Huang R. Sun T. (2022). Game-based learning for learners with disabilities—What is next? A systematic literature review from the activity theory perspective. *Front. Psychol.* 12:814691. 10.3389/fpsyg.2021.814691 35211058 PMC8861503

[B73] Tlili A. Lin V. Chen N.-S. Huang R. Kinshuk (2020). A systematic review on robot-assisted special education from the activity theory perspective. *Educ. Technol. Soc.* 23 95–109.

[B74] Vygotsky L. S. Cole M. (1978). *Mind in society: Development of higher psychological processes*. Cambridge, MA: Harvard University Press.

[B75] Wallis S. (2015). Structures of logic in policy and theory: Identifying sub-systemic bricks for investigating, building, and understanding conceptual systems. *Found. Sci.* 20 213–232. 10.1007/s10699-014-9360-4

[B76] Wang S. Sauer S. J. Schryver T. (2019). The benefits of early diverse and late shared task cognition. *Small Group Res.* 50 408–440. 10.1177/1046496419835917

[B77] Wong E. M. Ormiston M. E. Tetlock P. E. (2011). The effects of top management team integrative complexity and decentralized decision making on corporate social performance. *Acad. Manag. J.* 54 1207–1228. 10.5465/amj.2008.0762

[B78] Yates F. Oliveira S. (2016). Culture and decision making. *Organ. Behav. Hum. Decis. Process.* 136 106–118. 10.1016/j.obhdp.2016.05.003 32288179 PMC7126161

[B79] Yeung N. Summerfield C. (2012). Metacognition in human decision-making: Confidence and error monitoring review. *Natl Library Med.* 367 1310–1321. 10.1098/rstb.2011.0416 22492749 PMC3318764

[B80] Zhang Y. Waldman D. Yu-Lan H. Xiao-Bei L. (2015). Paradoxical leader behaviors in people management: Antecedents and consequences. *Acad. Manag. J.* 58 538–567. 10.5465/amj.2012.0995

[B81] Zhou K. Ariello L. M. Scepanovic S. Quercia D. Konrath S. (2021). The language of situational empathy. *Proc. ACM Hum. Comput. Interact.* 5 1–19. 10.1145/344908736644216

[B82] Zhou W. Zhu Z. Vrendenburgh D. (2020). Emotional intelligence, psychological safety, and team decision making. *Team Perform. Manag.* 26 123–142. 10.1108/TPM-10-2019-0105

[B83] Ziv G. (2011). Cognitive structure and foreign policy change: Israel’s decision to talk to the PLO. *Int. Relat*. 25, 426–454. 10.1177/0047117811404580

